# Use of health care services among Syrian refugees migrating to Norway: a prospective longitudinal study

**DOI:** 10.1186/s12913-021-06571-5

**Published:** 2021-06-10

**Authors:** Jasmin Haj-Younes, Elisabeth Marie Strømme, Jannicke Igland, Eirik Abildsnes, Bernadette Kumar, Wegdan Hasha, Esperanza Diaz

**Affiliations:** 1 Department of Global Public Health and Primary Care, University of Bergen, PO Box 7804, 5020 Bergen, Norway; 2 Department of Psychosocial Health, University of Agder, PO Box 422, 4604 Kristiansand, Norway; 3 Unit for Migration and health, Norwegian Institute of Public Health, PO Box 222, 0213 Oslo, Norway

**Keywords:** Refugees, Migrants and Transients, Health services utilization, Longitudinal

## Abstract

**Background:**

Understanding the differential utilization of healthcare services is essential to address the public health challenges. Through the migration process, refugees move from one set of health risk factors to another and can face multiple healthcare challenges along their journey. Yet how these changing risk factors influence refugees’ use of health care services is poorly understood.

**Methods:**

A longitudinal survey assessing health care utilization of 353 adult Syrian refugees was conducted; first in a transit setting in Lebanon and after one year of resettlement in Norway. The main outcomes are the utilization of general practitioner services, emergency care, outpatient and/or specialist care and hospitalization during the previous 12 months. Associations between use of healthcare services and several sociodemographic, migration-related and health status variables at both time points were found using regression analysis. We also analyzed longitudinal changes in utilization rates using generalized estimating equations.

**Results:**

The use of general practitioner and emergency care increased after resettlement while outpatient/specialist care markedly dropped, and hospitalization rates remained the same. Undocumented status and poor self-rated health (SRH) prior to resettlement were identified as predictors for use of health care after arrival. After resettlement, higher health literacy, higher education, higher social support and poor SRH and quality of life were significantly associated with use of healthcare services.

**Conclusions:**

Utilization of health services changes post migration to the destination country and are associated with migration-related and socio-demographic factors. Poor SRH is associated with use of services, both pre-arrival and post-resettlement. Our findings have implications for future resettlements, health care policies and service provision to newly arrived refugees with regard to both health needs as well as delivery of services.

**Supplementary Information:**

The online version contains supplementary material available at 10.1186/s12913-021-06571-5.

## Introduction

Many countries in Europe have long humanitarian tradition of receiving and resettling forcibly displaced individuals [1] and should provide equitable healthcare services to an increasingly diverse population [2]. Responding to changes in demographics and attaining equity in health can be viewed as a public health investment. However, for many European countries this is hampered by the lack of reliable knowledge of the health status and health needs of forced migrants in the early phase of resettlement [3]. Without adequate information, many resettlement-countries are unable to assess whether services are accessible for forced migrants and if needs are efficiently met [2].

The utilization of healthcare services is a multidimensional process that combines need for, and access to care. In an optimal scenario, use of services should be proportional to ones need [4]. Even though access of and use of services are inter-related, they are distinct parts of the health delivery process where utilization presumes access [5]. Factors related to access to services have been conceptualized in many ways, and commonly includes aspects on both the provider side and the user side such as accessibility, affordability, availability and appropriateness [6]. Both access and use of healthcare services is hence influenced by context, meaning that even where entitlements are formally established and financial barriers are lifted, access and use are influenced by resources required for good health, such as social support, education, and health literacy. Likewise, one might argue that additional factors related to the migration experience affect the use of health care services for forced migrants given the risk of exposure to external factors such as persecution, food insecurity, and violence. Exposures that can shape the forced migrants’ health profile and subsequently their need for care [7]. This, however, has scarcely been researched.

The Syrian refugee crisis remains the largest displacement crisis in the world, with 5.6 million registered refugees seeking transient safety in neighboring countries [8]. While in transit, healthcare services are often characterized by high privatization, fragmented between many different providers, making access to care difficult and costly [9]. For undocumented migrants, economic barriers are further aggravated with fear of detention or deportation if seeking healthcare [9]. Upon arrival in Norway, refugees are invited to a general health assessment, and have the same rights and entitlements to services as the resettlement country population. The Norwegian health care system offers universal coverage with relatively small out-of-pocket expenses. The general practitioner (GP) serves as a gatekeeper to secondary care, regulating the access to specialist and hospital care [10]. Primary care services are thus patient-driven while influx into secondary care is managed by healthcare providers.

Through the resettlement process, refugees move from one set of health risk factors to another and can face multiple additional healthcare challenges along their journey. Few studies have focused on this change of context and environment, how it affects subsequent use of health care and whether adverse conditions affecting health and the use of health care services pre-arrival persists post migration. Applying a longitudinal design allowing a trajectory perspective, our study aimed to: (a) describe patterns of health care service use in Lebanon and Norway, (b) identify pre-arrival sociodemographic and migration-related predictors of health care service use post migration and (c) identify post-arrival factors associated with health care service use in the resettlement country.

## Methods

### Study design, participants, and data collection

This is a two-time points follow-up study which is part of the *Changing Health and health care needs Along the Syrian Refugees’ Trajectories to Norway* (CHART) project [11], assessing health of Syrian refugees in Norway. Methods were carried out in accordance with the Strengthening the Reporting of Observational Studies in Epidemiology (STROBE) Statement guidelines and with national and European privacy legislation.

In this paper, we focus on persons recognized as refugees by the United Nations High Commissioner for Refugees (UNHCR) accepted for third-country resettlement [1]. The methods have already been described elsewhere [12]. In brief, a baseline self-administered survey was conducted in Lebanon in 2017–2018, followed by a follow-up survey in Norway after one year. A total of 514 Syrian nationals from 16 and above attending the mandatory Norwegian Cultural Orientation Programme (NORCO) in the given period were included in the study in Lebanon. The Arabic baseline questionnaire was distributed during course time under the guidance of cross-culturally responsive bilingual trainers. Follow-up measurements post-arrival were gathered through structured telephone interviews in Arabic. A total of 506 eligible subjects completed the baseline survey (98 %), of which 464 (92 %) were confirmed resettled in Norway and 353 completed the second questionnaire (70 %) (Supplementary Fig. 1).

### Dependent variables

The main outcomes for this study are the utilization of a GP, emergency care (EC), outpatient and/or specialist care as well as hospitalization during the previous 12 months. These four main outcomes were assessed through the following questions: ‘During the last 12 months, have you visited any of the following: a general practitioner, emergency care, outpatient care, specialist care (yes/no)’ and ‘Have you been admitted to the hospital the last 12 months? (yes/no)’. Given similarities in outpatient and specialist care in Norway, where the main point is to be assessed by a medical specialist, these two variables were merged into one. The two items are based on questions from The Nord-Trøndelag Health Study (HUNT) [13].

### Independent variables

Self-rated health (SRH) was measured as an indicator of the need for healthcare at both time points. We used a validated single-item question: “How do you consider your health at the moment?”, with a five-point Likert scale ranging from very poor to very good. The item was dichotomized merging ‘very poor’ and ‘poor’ indicating poor SRH versus non-poor SRH. The SRH-item has shown acceptable validity and reliability among Arabic speakers and in refugee populations [14, 15]. Additionally, we measured quality of life (QoL) using the WHO Quality of Life Scale (WHOQOL-BREF), a transcultural instrument previously validated in Arabic [16], which includes a total of 26 questions on physical health, mental health, social relationships, and environment [17]. Each item is rated on a five-point Likert scale with a higher score indicating a better QoL. Raw scores were transformed creating domain scores within the range of 4–20 by multiplying the average of the items in each domain by four, in accordance with the user’s manual [17].

Perceived social support was measured with the 7-item ENRICHD Social Support Instrument (ESSI) [18]. A total score is the sum of all items with higher scores indicating better social support. A binary measure for high social support defined as having answered > 2 on at least two items and a total score of > 18 was created, based on the definition of low-social support [18]. ESSI has previously been validated among Syrian refugees [19].

Sociodemographic variables encompassed age, gender, primary language spoken, marital status and level of education. In addition, we assessed Health Literacy through the single-item literacy screener (SILS): “How often do you need help reading written material from your doctor or pharmacy?” With a five-point Likert scale. Scores higher than 2 point to difficulties with reading health-related material. We also inquired on migration-related factors such as time since the flight from Syria, migrating alone or with family, residence permit in Lebanon, and possible exposure to traumatic events with The Single General Trauma Item [20].

The entire questionnaire was in Arabic; it contained questions already translated and validated and those sections that were not went through a standardized translation process [21].

### Statistical analysis

We present sociodemographic and migration-related characteristics as counts and proportions for categorical variables, medians, and interquartile ranges (IQR), and means and standard deviations (SD) for continuous variables (Table 1). Selection bias between the cohort and the loss-to-follow-up group was assessed using *χ*^2^-statistics and independent group’s *t*-tests (Supplementary Table 1).

**Fig. 1:**
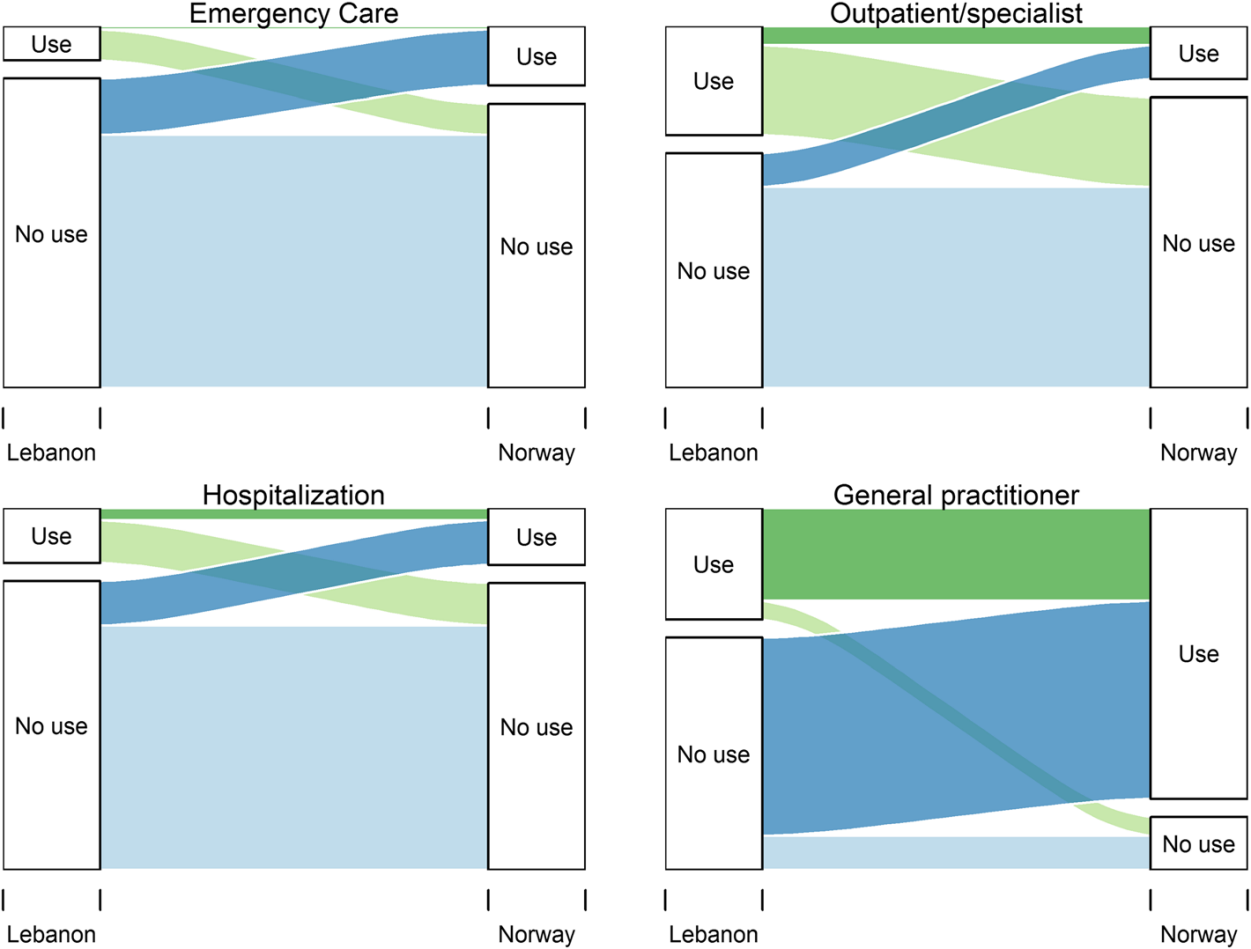
Trajectories of healthcare utilization from baseline to follow-up

We used a Sankey chart to visualize the changes in use of services before arrival and after resettlement by creating trajectory variables with the proportions going from use to no use and vice versa or no change in outcomes (Fig. 1). Changes in the use of health services from baseline to follow-up were also analyzed using generalized estimating equations (GEE) with data in long format with two observations per individual and “wave” as a binary covariate (Table 2). We applied a log-link and binomial distribution and reported exponentiated regression coefficients as risk ratios (RR) with 95 % CI.

We used multivariate analysis to evaluate factors associated with the use of healthcare services in Norway looking at selected sociodemographic and migration-related factors as well as self-perceived health status and QoL at baseline and follow-up. First, we looked at baseline characteristics in Lebanon as predictors for the use of services after arrival in Norway. Thereafter, we looked at characteristics while in Norway and associations with the use of services in Norway. We used log-binomial regression analysis reported as risk ratios with 95 % confidence intervals in two models; (1) unadjusted (2) adjusted for potential confounders for the total effect of each characteristics on the outcome based on results from a directed acyclic graph (DAG) depicted in supplementary Fig. 2. The DAG was constructed using the software DAGitty [22]. For instance, for the total effect of health literacy at baseline on use of health services in Norway, age, gender, and education were potential confounders, while SRH at baseline was considered as a mediator and not adjusted for. In cases where convergence was not achieved in log-binomial regression analysis, Poisson regression was used with robust error variance (Table 3) [23].

Missing values were handled through listwise deletions. An alpha value of 0.05 was considered statistically significant. We analyzed the data using STATA/IC software, version 16.0, (StataCorp LLC, Texas, USA).

## Results

### Characteristics of the study population

Sociodemographic characteristics and self-perceived health and QoL of this cohort has been published elsewhere but are stated in Table 1 for the sake of clarity. We included data from 353 participants in the final analysis (supplementary Fig. 1). The respondents did not differ from the loss-to-follow-up group in terms of age or gender but had higher health literacy (supplementary Table 1).

**Table 1:** Sociodemographic and migration related factors, *N* = 353

SOCIODEMOGRAPHIC FACTORS	BASELINE	FOLLOW-UP
Gender (n, %)		
Women	181 (51)	-
Men	171 (49)	-
Age in years (median, IQR)	34 (27–41)	-
Native tongue (n, %)		
Arabic	335 (95)	-
Kurmanji	15 (4)	-
Marital status (n, %)		
Married	265 (75)	260 (75)
Number of children (median, IQR)	3 (2–4)	3 (2–4)
Education in years (median, IQR)	8 (6–10)	8 (6–9)
High health literacy^a^ (n, %)	195 (56)	23 (7)
High social support^b^ (n, %)	123 (35)	210 (60)
**HEALTH AND QUALITY OF LIFE**		
Good Self-rated health (n, %)	203 (58)	221 (63)
Poor self-rated health (n, %)	67 (19)	51(15)
Physical health (WHOQOL-BREF domain 1) (mean, SD)	13.7 (2.7)	15.6 (2.8)
Psychological health (WHOQOL-BREF domain 2) (mean, SD)	12.8 (2.7)	14.5 (2.3)
Social relationships (WHOQOL-BREF domain 3) (mean, SD)	13.7 (2.9)	15.3 (2.8)
Environment (WHOQOL-BREF domain 4) (mean, SD)	8.9 (2.4)	14.0 (2.2)
**MIGRATION RELATED FACTORS**		
Time since flight from Syria at baseline in years (median, IQR)	5 (4–6)	-
Time since arrival in Lebanon at baseline in years (median, IQR)	5 (4–5)	-
Been in other transit country before Lebanon (n, %)	20 (6)	-
No residence permit in Lebanon at baseline (n, %)	242 (69)	-
Migrating alone to Lebanon (n, %)	55 (16)	-
Length of stay in Norway at follow-up in months (median, IQR)	-	14 (12–15)
Experience of pre-migration trauma (n, %)	135 (40)	-

^a^High health literacy defined as scores ≤ 2 (Likert scale from 1 to 5). ^b^High social support defined as > 2 on at least two of the seven ESSI items and a total score of > 18, range for ESSI 0–22.

### Use of healthcare services and changes in use from Lebanon to Norway

Of the 353 participants, 33 % visited a GP in Lebanon, 32 % visited outpatient/specialist care, 16 % were hospitalized, and 10 % used EC in the 12 preceding months at baseline (Table 2). In Norway, the use of a GP increased to 85 % and the use of EC to 18 % while hospitalizations remained the same and outpatient/specialist care visits dropped to 16 %. In Fig. 1, we present Sankey charts showing trajectories of healthcare service use. Most participants did not use EC, outpatient/specialist care, or hospital care neither at baseline nor at follow-up. There were 16 % new reports of EC use at follow-up, while 9 % used this in Lebanon but not in Norway. For specialist/outpatient care, 10 % reported new use while 26 % reported using this in Lebanon but not in Norway. The biggest change in trajectory is the increase in the use of GP from pre-arrival to after resettlement with 58 % new reports of use.

**Table 2:** Changes in healthcare service utilization from Lebanon to Norway

	Baseline		Follow-up		Change	
	N	n (%)	N	n (%)	RR (CI)	p-value
**Variable**						
General Practitioner (yes)	345	112 (33)	353	300 (85)	2.6 (2.2–3.1)	< 0.001
Emergency care (yes)	343	34 (10)	352	62 (18)	1.7 (1.2, 2.7)	0.005
Outpatient/Specialist (yes)	346	109 (32)	353	55 (16)	0.5 (0.4, 0.7)	< 0.001
Hospital (yes)	346	55 (16)	352	56 (16)	1.0 (0.7, 1.4)	0.991

Abbreviations: RR = Relative risk. CI = Confidence interval.

### Pre-arrival predictors of use of health care services in Norway

Increasing age was significantly associated with the use of EC services and hospitalization after arrival (Table 3). No other significant associations between pre-arrival sociodemographic factors and the use of healthcare services at follow-up were found. With regards to health status pre-arrival, we found that poor SRH was significantly associated with increased risk of EC use after arrival, while lower scores in the social relationships’ domain of QoL (i.e., poorer social relationships) were significantly associated with use of EC after arrival. With regards to migration-related factors, not having a residence permit in the transit country was significantly associated with the use of EC after arrival.

**Table 3:** Associations between sociodemographic characteristics, health status and migration related factors at baseline and follow-up and use of emergency care, outpatient/specialist services and hospitalizations at follow-up

	General Practitioner at T2		Emergency care at T2		Outpatient/Specialist care at T2		Hospitalization at T2	
	RR (CI 95 %)	ARR (CI 95 %)	RR (CI 95 %)	ARR (CI 95 %)	RR (CI 95 %)	ARR (CI 95 %)	RR (CI 95 %)	ARR (CI 95 %)
**Sociodemographic factors at T1**								
*Gender*								
Male (reference)	1	1	1	1	1	1	1	1
Female^A^	1.09 (0.99, 1.19)	1.09 (0.99, 1.19)	1.10 (0.70, 1.73)	1.34 (0.83, 2.08)	0.84 (0.52, 1.37)	0.81 (0.50, 1.33)	0.85 (0.53, 1.36)	1.01 (0.62, 1.64)
Age (years)^B^	1.00 (0.99, 1.00)	1.00 (0.99, 1.00)	1.03 (1.01, 1.05)**	1.04 (1.02, 1.06)**	1.01 (0.98, 1.03)	1.01 (0.98, 1.03)	1.03 (1.01, 1.05)*	1.03 (1.01, 1.05)*
Education (continuous)^C^	0.99 (0.99, 1.01)	1.00 (0.99, 1.01)	0.93 (0.87, 1.00)*	0.95 (0.90, 1.01)	0.98 (0.91, 1.06)	0.98 (0.91, 1.06)	0.96 (0.90, 1.04)	0.98 (0.92, 1.05)
Health literacy (continuous)^D^	1.01 (0.98, 1.05)	1.01 (0.98, 1.05)	0.99 (0.84, 1.18)	0.89 (0.73, 1.10)	0.95 (0.79, 1.15)	0.91 (0.74, 1.13)	1.07 (0.90, 1.27)	0.97 (0.80, 1.19)
*Social support (categorical)*								
Low social support (reference)	1	1	1	1	1	1	1	1
High social support^C^	1.02 (0.92, 1.12)	1.00 (0.91, 1.11)	1.18 (0.72, 1.93)	1.46 (0.86, 2.47)	1.24 (0.73, 2.13)	1.35 (0.77, 2.35)	1.22 (0.72, 2.05)	1.56 (0.90, 2.70)
**Health status and Quality of life at T1**								
*Self-rated health (categorical)*								
Moderate to good SRH (reference)	1	1	1	1	1	1	1	1
Poor SRH^E^	1.03 (0.92, 1.14)	1.05 (0.95, 1.16)	1.72 (1.06, 2.77)*	1.72 (1.06, 2.78)*	1.43 (0.83, 2.47)	1.36 (0.77, 2.43)	1.17 (0.65, 2.08)	1.05 (0.60, 1.84)
*Quality of life (continuous)*								
Physical health (WHOQOL-BREF domain 1)^E^	0.99 (0.99, 1.00)	0.99 (0.97, 1.01)	0.98 (0.90, 1.06)	0.99 (0.90, 1.08)	0.96 (0.88, 1.05)	0.96 (0.88, 1.06)	0.98 (0.90, 1.07)	0.99 (0.91, 1.09)
Psychological health (WHOQOL-BREF domain 2)^E^	1.00 (0.99, 1.02)	1.00 (0.99, 1.02)	1.04 (0.95, 1.13)	1.05 (0.96, 1.15)	0.99 (0.91, 1.09)	0.99 (0.90, 1.09)	0.99 (0.90, 1.08)	1.00 (0.91, 1.11)
Social relationships (WHOQOL-BREF domain 3)^E^	0.99 (0.98, 1.00)	0.99 (0.98, 1.01)	0.94 (0.88, 1.01)	0.93 (0.88, 0.99)*	1.04 (0.95, 1.13)	1.06 (0.96, 1.16)	0.94 (0.88, 1.01)	0.95 (0.89, 1.01)
Environment (WHOQOL-BREF domain 4)^E^	0.99 (0.97, 1.00)	0.99 (0.97, 1.01)	0.96 (0.88, 1.06)	0.98 (0.89, 1.08)	1.03 (0.93, 1.13)	1.05 (0.94, 1.17)	0.89 (0.80, 0.99)*	0.93 (0.84, 1.03)
**Migration related factors**								
Time since flight from Syria (continuous)^C^	0.98 (0.97, 0.98)*	0.97 (0.94, 1.01)	0.97 (0.80, 1.17)	0.94 (0.80, 1.11)	0.99 (0.80, 1.23)	0.98 (0.80, 1.22)	1.01 (0.81, 1.26)	0.97 (0.79, 1.20)
No residence permit in Lebanon^C^	1.12 (1.00, 1.25)*	1.10 (0.99, 1.25)	1.95 (1.06, 3.60)*	2.72 (1.39, 5.31)**	1.05 (0.62, 1.81)	1.04 (0.61, 1.79)	0.62 (0.38, 1.00)	0.74 (0.45, 1.22)
Migrating alone to Lebanon^C^	0.66 (0.38, 1.13)	0.67 (0.39, 1.16)	1.25 (0.32, 4.83)	0.64 (0.24, 1.72)	1.16 (0.45, 3.01)	3.15 (0.64, 15.4)	1.31 (0.59, 2.92)	1.04 (0.54, 2.07)
Trauma exposure before resettlement in Norway^C^	0.96 (0.88, 1.05)	1.05 (0.96, 1.15)	0.77 (0.48, 1.24)	1.49 (0.95, 2.32)	0.84 (0.51, 1.39)	1.16 (0.69, 1.93)	0.57 (0.34, 0.94)*	1.63 (0.98, 2.71)
**Sociodemographic factors at T2**								
*Gender*								
Male (reference)	1	1	1	1	1	1	1	1
Female^A^	1.06 (0.97, 1.16)	1.06 (0.97, 1.16)	1.13 (0.71, 1.80)	1.28 (0.81, 2.00)	0.86 (0.52, 1.42)	0.88 (0.53, 1.46)	0.86 (0.53, 1.41)	0.96 (0.59, 1.57)
Age (years)^B^	1.00 (0.99, 1.00)	1.00 (0.99, 1.00)	1.03 (1.01, 1.05)**	1.04 (1.02, 1.06)**	1.01 (0.99, 1.03)	1.01 (0.99, 1.03)	1.03 (1.01, 1.05)**	1.03 (1.01, 1.05)**
Education (continuous)^F^	1.00 (0.99, 1.01)	1.02 (0.99, 1.04)	0.96 (0.90, 1.02)	1.05 (0.92, 1.19)	1.02 (0.96, 1.10)	1.11 (0.99, 1.25)	1.03 (0.96, 1.10)	1.19 (1.08, 1.31)**
Health literacy (continuous)^G^	1.07 (1.03, 1.12)*	1.06 (1.02, 1.1)*	1.37 (1.08, 1.74)*	1.28 (1.02, 1.61)*	1.24 (0.96, 1.60)	1.21 (0.93, 1.59)	1.61 (1.25, 2.06)**	1.59 (1.25, 2.01)**
*Social support (categorical)*								
Low social support (reference)	1	1	1	1	1	1	1	1
High social support^H^	1.02 (0.93, 1.12)	1.04 (0.94, 1.14)	1.69 (1.02, 2.80)*	1.82 (1.11, 2.98)*	1.82 (1.05, 3.18)*	1.83 (1.04, 3.24)*	1.75 (1.02, 3.00)*	1.83 (1.06, 3.16)*
**Health status and Quality of life at T2**								
*Self-rated health (categorical)*								
Moderate to good SRH (reference)	1	1	1	1	1	1	1	1
Poor SRH^I^	0.99 (0.87, 1.13)	0.99 (0.86, 1.14)	2.17 (1.35, 3.47)*	1.92 (1.15, 3.20)*	1.81 (1.05, 3.13)	1.88 (1.03, 3.43)*	2.93 (1.84, 4.66)**	2.49 (1.49, 4.15)**
*Quality of life (continuous)*								
Physical health (WHOQOL-BREF domain 1)^I^	1.00 (0.98, 1.01)	0.99 (0.98, 1.01)	0.90 (0.85, 0.94)**	0.91 (0.84, 0.97)**	0.90 (0.85, 0.96)*	0.89 (0.84, 0.95)**	0.87 (0.83, 0.92)**	0.88 (0.82, 0.94)**
Psychological health (WHOQOL-BREF domain 2)^I^	1.02 (1.00, 1.04)*	1.02 (0.99, 1.04)	0.87 (0.81, 0.93)*	0.89 (0.84, 0.98)*	0.94 (0.85, 1.04)	0.96 (0.84, 1.09)	0.89 (0.82, 0.98)*	0.89 (0.80, 0.99)*
Social relationships (WHOQOL-BREF domain 3)^I^	0.99 (0.98, 1.01)	1.00 (0.98, 1.01)	0.91 (0.86, 0.96)*	0.93 (0.86, 1.00)	0.92 (0.86, 0.99)*	0.90 (0.86, 0.99)*	0.88 (0.83, 0.93)**	0.90 (0.84, 0.97)**
Environment (WHOQOL-BREF domain 4)^I^	1.04 (1.02, 1.05)**	1.04 (1.02, 1.06)**	1.06 (0.96, 1.17)	1.08 (0.96, 1.21)	1.03 (0.93, 1.15)	1.01 (0.89, 1.15)	1.06 (0.96, 1.18)	1.06 (0.91, 1.23)

A: adjusted for age. B: adjusted for gender. C: adjusted for age and gender. D: adjusted for age, gender, social support at T1 and education at T1. E: adjusted for age, gender, social support at T1 and trauma exposure. F: adjusted for age, gender and education at T1. G: adjusted for age, gender, social support at T1, education at T1 and health literacy at T1. H: adjusted for age, gender, social support at T1 and trauma exposure. I: adjusted for age, gender, social support at T1, SRH and QoL at T1, time since flight from Syria, no residence permit at T1 and trauma exposure. Significant results with *P* < 0.05 are marked with an asterisk. Significant results with *P* < 0.01 are marked with two asterisks

### After-arrival factors associated with the use of health care services in Norway

When in Norway, increasing age was still significantly associated with use of EC services and hospitalization (Table 3). Likewise, increased health literacy was significantly associated with use of GP, EC and hospitalization. Similarly, high social support (ESSI) was significantly associated with increased risk of EC use, use of outpatient/specialist care and hospitalization, and increasing education level was associated with hospitalization. When looking at health status, we found that poor SRH was significantly associated with the use of both EC and hospitalizations. Generally, lower scores in the different QoL dimensions were associated with higher use of services. However, higher scores in the environmental domain of QoL were significantly associated with use of a GP.

## Discussion

This study provides data on health care utilization before and after resettlement assessed at two different locations and time points following the journeys of the same participants and therefore incorporates factors from the pre-arrival context as possible predictors for later use. We find that not having a residence permit and having poor health status pre-arrival predict the use of services after resettlement. Poor SRH was significantly associated with use of services both in Lebanon and in Norway suggesting a stable association along the migration path. For the post migration stage, we find a significant association between the use of healthcare services and increasing health literacy (SILS), high social support (ESSI), education and poor QoL. These factors did not seem to influence future health care behavior while in transit, suggesting phenomena subjected to change with time and context. Also, we find an increase in GP and EC use after resettlement and a decrease in outpatient/specialist care while hospitalization rates do not change pre- and post-resettlement, probably mirroring the health care system in the country of stay at each period.

Use of GP services more than doubled pre- and post-resettlement. This rate (85 %) is slightly higher than that of the resettlement country population in Norway, where 75 % reported use of GP in the last 12 months in population-based data [24]. Comparing numbers between surveys is encumbered with uncertainties, but we believe some of the differences in GP utilization between our sample and the resettlement country population can be explained by the fact that in some Norwegian municipalities, the general health assessment upon arrival is performed by a GP. Despite having a separate question for the general health assessment, we assume some participants might have had difficulties distinguishing between the two alternatives as both entails contact with a primary care doctor. Another possibility is that some refugees were derived to a second visit by the GP at the first encounter for their general health assessment. In any case, it is important to acknowledge the key opportunity GPs have in responding to the need of the refugee patient in early resettlement as the first point of contact. Previous studies have argued that refugee primary care services might reduce unnecessary EC use [25], showing that refugees who receive a health assessment shortly after arrival will be less likely to have an acute care visit in this period [26]. Furthermore, we found an increase in EC use from 10 % before arrival to 16 % after resettlement, which is similar to the utilization rates of the resettlement country population [27].

On the other hand, the use of outpatient/specialist care dropped from 32 % in Lebanon to 16 % in Norway. This decrease might be explained at the system level, since outpatient/specialist care services in Norway require a referral, usually from a GP, while other routes are available to access such care in Lebanon given a highly privatized health sector. When comparing with population-based data from the Norwegian population, 36 % reported having had contact with outpatient/specialist care the last 12 months [24]. A number twice as high as that of our population, but not adjusted for morbidity, so potential under-or overuse is not possible to determine with certainty. Furthermore, some of our respondents might have been referred by their GPs to secondary care, but still waiting for their appointments with a specialist at the time of the follow-up survey. However, the doctor-patient interaction is key in identifying patients needing a referral [28]. Previous research has shown that not speaking the same language is associated with decreased symptom reporting, fewer referrals to specialist care [29] and shorter consultation time [30], which also could explain our results. An inverse socioeconomic gradient in terms of utilization of outpatient/specialist care has also been documented in Norway [31] that confirms privileged groups are those that avail most of services [32]. However, utilization of GP and hospital admissions, which is easier to access, was found to be equitable [33]. Similarly, a systematic review across Europe showed that outpatient visits for specialized care were generally used less often by migrants [34]. In our sample, hospital admissions did not change pre- and post-resettlement, which could point to hospital admissions having similar access thresholds across countries.

Finding pre-migration predictors for use of health care in Norway can be of key importance to adequately prepare health services to the new migrant population. One novel finding in this study is that not having a residence permit in the transit country and having poor social relationships in transit was associated with higher use of emergency care the first year after resettlement. Generally, the lack of recognized documentation in a country complicates the availability of healthcare and one can assume that acute and/or chronic diseases left uncared for contribute to higher use of care post-resettlement. Hence, securing minimum acceptable living conditions for refugees in transit countries should be a priority concern globally.

The strongest correlation we found was the one between poor SRH and health care utilization, signifying the concordance between need for care and use of care. Perceived poor health status seems to be a stable factor as it holds for both pre-arrival health status and after resettlement, even though the association after resettlement is stronger. While the association between health need and health care utilization is well-known [35], our study highlights the stability of this association along the migration trajectory. Post-migration, we found associations between use of services and higher health literacy, higher education, higher social support (ESSI), and low levels of QoL. High health literacy drops from 56 % in Lebanon to only 7 % in Norway, pointing to challenges with a new language and a different health care system, while high social support (ESSI) somewhat unexpectedly increases from 35 % in Lebanon to 60 % in Norway. This increase might be explained by the fact that most quota refugees are resettled as families and some are re-united with extended family members preceding them to the resettlement country. Easier access to online communication and established support networks upon arrival can also explain this increase. Why persons with high health literacy, higher education and high social support have increased probability of use while in Norway but not in transit is difficult to answer but we assume these factors become more important in a context where there is universal health coverage, and no economic barriers to health care.

For the concept of social support and social relationships, we found associations pointing in opposite directions. Poor social relationships measured with WHOQOL-BREF while in Lebanon was associated with use of EC after arrival. When in Norway, high social support (ESSI) was associated with use of EC, outpatient/specialist care and hospitalizations. Likewise, poor social relationships (WHOQOL-BREF) were associated with outpatient/specialist care and hospitalizations. We believe some of this can be explained by measurement differences in social support instruments, not capturing the exact same phenomenon. The social relationships domain in WHOQOL-BREF as part of QoL only consists of three questions (satisfaction with relationships, satisfaction with support from friends and satisfaction with sexual relationships) and has the concept of satisfaction in it while ESSI consist of 7 questions and asks directly if you have someone available to talk to, receive advice, emotional support, receive help with daily chores etc. without assessing satisfaction.

The environmental domain of QoL describes feeling of safety, satisfaction of living place, enough money to meet needs, and satisfaction with transportation. Interestingly, we found that higher scores in this domain were associated with use of a GP. This also confirms the inverse care law [32].

## Strengths and limitations

Working with a cohort with similar background arriving at the same time minimizing influence of contextual factors as well as a high response rate and the use of validated instruments add to the strengths of this study. However, certain limitations need to be considered when interpreting our data. We did not assess frequencies of contact with the healthcare services, only yes/no for use at least once. Because of this we are not able to separate between frequent users and persons who have only used the service once. This study has an explorative nature with a high number of statistical tests, which increases the risk of Type 1 error. We can therefore not rule out that some of the significant results are chance findings, especially those with p-values close to 0.05 (marked with one asterisk in Table 2). In addition, the variable health literacy is assessed with only one question (SILS) which is limited and has to our knowledge not been validated in a refugee population with poor language skills upon resettlement. Moreover, we deliberately changed mode of data collection from self-completion to structured interviews between the two time points which can introduce a possibility of interviewer bias, but in that way, we achieved a high response rate. Further, we should ideally have had a longer follow-up time to better assess changes with time. However, previous research has highlighted that we especially lack data on the first 5 years after resettlement [36]. Last, utilization of care is not equal with appropriate care or equality in quality of care, which we are unable to evaluate with the current study design.

Despite these limitations, we believe our findings add important knowledge to the field of health services research for refugees, a group that is understudied in health system research. Based on our findings, we encourage resettlement countries to enhance primary care services in providing diversity-sensitive care given their role as first port of call. Possible under-use of specialist/outpatient care among refugees and reasons for such differences warrants further research. People with undocumented status before arrival should be subjected to extra awareness to secure healthcare needs being effectively met at the primary care level. Social support and health literacy can be possible targets for future interventions to enhance accessibility of care. In conclusion, the use of healthcare for refugees clearly changes from the pre-and-post resettlement phase. Apart from entitlements and need, health care utilization is impacted by sociodemographic factors and migration-related factors.

## Supplementary information

**Additional file 1**

## Data Availability

The datasets generated and/or analysed during the current study are not publicly available due to data protection regulations in Norway but are available from the corresponding author on reasonable request.

## Abbreviations

CHART: Changing Health and health care needs Along the Syrian Refugees’ Trajectories to Norway
DAG: Directed Acyclic Graph
EC: Emergency Care
ESSI: ENRICHD Social Support Instrument
GEE: Generalized Estimating Equations
GP: General practitioner
HUNT: Nord-Trøndelag Health Study
NORCO: Norwegian Cultural Orientation Programme
SRH: Self-rated Health
QoL: Quality of Life
UNHCR: United Nations High Commissioner for Refugees
